# Audiologisches Ergebnis bimodal versorgter CI-Träger:innen im zeitlichen Verlauf und in Abhängigkeit unterschiedlicher Einflussfaktoren

**DOI:** 10.1007/s00106-024-01508-w

**Published:** 2024-08-27

**Authors:** Hanna Schlegel, S. Hartmann, S. Kreikemeier, E. Dalhoff, H. Löwenheim, A. Tropitzsch

**Affiliations:** 1 Universitätsklinik für Hals‑, Nasen- und Ohrenheilkunde, Eberhard Karls Universität Tübingen, Elfriede-Aulhorn-Straße 5, 72076 Tübingen, Deutschland; 2 Fachbereich Akustik und Audiologie, Hochschule Aalen, Aalen, Deutschland; 3 Helios Hörklinik Oberbayern, Steinerweg 5, 81241 München, Deutschland

**Keywords:** Cochleaimplantat, Asymmetrischer Hörverlust, Bimodale Hörversorgung, Sprachverstehen, Indikation, Cochlear implant, Asymmetric hearing loss, Bimodal hearing, Speech comprehension, Indication

## Abstract

**Hintergrund:**

Hörgeschädigte Menschen mit asymmetrischem Hörverlust und einseitiger Indikation für ein Cochleaimplantat (CI) profitieren in aller Regel deutlich von einer bimodalen Hörversorgung. Der Einfluss dieser Versorgungsart auf das Sprachverstehen (SV) im zeitlichen Verlauf ist bislang nicht hinreichend untersucht. Die vorliegende Studie untersucht den Einfluss einer bimodalen Versorgung auf das SV postlingual ertaubter, bimodal versorgter CI-Träger:innen nach einer Tragedauer von mindestens 36 Monaten und analysiert dabei mögliche Einflussfaktoren.

**Methode:**

Es wurden 54 bimodal versorgte lautsprachkompetente CI-Träger:innen mit einer CI-Erfahrung von mindestens 36 Monaten in diese retrospektive Längsschnittstudie eingeschlossen. Audiometrische Daten von diesen CI-Träger:innen wurden im zeitlichen Verlauf verglichen.

**Ergebnisse:**

Die Veränderung der Ergebnisse im Freiburger Einsilbertest (FBE) im Verlauf der 36 Monate war für die Ertaubungsgruppe < 10 Jahre sowohl für den Pegel 65 dB „sound pressure level“ (SPL) als auch für 80 dB SPL signifikant und für die Ertaubungsgruppe ≥ 10 Jahre für 65 dB SPL signifikant (*p* < 5 %). Beim Oldenburger Satztest (OlSa) ergab sich für die Konfigurationen S_0_, S_0_N_0_ und S_0_N_CI_ eine hochsignifikante Veränderung (*p* < 0,1 %). und für S_0_N_HG_ (HG: Hörgerät) eine sehr signifikante Veränderung (*p* < 1 %). Das Alter bei Versorgung als möglicher Einflussfaktor konnte durch den FBE nicht bestätigt werden. Die Ertaubungsdauer stellte dagegen einen negativen Einflussfaktor für das SV mit dem CI dar, wobei eine längere Ertaubungsdauer mit schlechteren Ergebnissen beim FBE assoziiert ist. Der Grad der Schwerhörigkeit des mit HG versorgten Ohrs beeinflusste das SV nicht. Im Median betrug der bimodale Nutzen (Differenz aus dem SV mit bimodaler Versorgung gegenüber einseitiger HG-Versorgung beim FBE für 65 dB SPL) über die gesamte Untersuchungszeit 10 %. Für im Median 79 % der Versuchspersonen war der bimodale Nutzen über den gesamten Zeitverlauf von 36 Monaten nachweisbar.

**Schlussfolgerung:**

Im zeitlichen Verlauf verbessert sich das SV mit dem CI der bimodalen Versuchspersonen signifikant. Die untersuchten Einflussfaktoren (Alter, Ertaubungsdauer und Grad der Schwerhörigkeit des Gegenohrs) unterstützen die leitliniengerechte Indikationsstellung einer bimodalen Versorgung in Deutschland – unabhängig von Alter, Ertaubungsdauer und Hörfähigkeit des Gegenohrs – eine Cochleaimplantation durchzuführen.

## Abstract (en)

**Background:**

Hearing-impaired persons with asymmetric hearing loss and a unilateral indication for a cochlear implant (CI) generally benefit from a bimodal hearing solution. The influence of bimodal fitting on speech comprehension (SC) over time has not yet been sufficiently investigated. The present study examines the influence of bimodal fitting on SC in bimodally fitted CI users with postlingual deafness at least 36 months after implantation and analyzes possible influencing factors.

**Methods:**

Included in this retrospective longitudinal study were 54 bimodally fitted speech-competent CI users with at least 36 months of CI experience. Audiometric data of these CI users at predefined timepoints were compared.

**Results:**

The change in the results of the Freiburg monosyllabic test (FT) over 36 months was significant (*p* < 5%) for the deafness group at <10 years for both the 65 dB sound pressure level (SPL) and at 80 dB SPL and also significant for the deafness group ≥10 years for 65 dB SPL. In the Oldenburg sentence test (OlSa) there was a highly significant change (*p* < 0.1%) for S_0_, S_0_N_0_, and S_0_N_CI_ configurations and a very significant change (*p* < 1%) for S_0_N_HA_ (HA: hearing aid). Age at implantation as a possible influencing factor could not be confirmed in the FT. In contrast, the duration of deafness was a negative influencing factor for SC with CI in the FT, whereas a longer duration of deafness was associated with worse results in the FT. The degree of hearing loss in the ear fitted with an HA did not influence SC. The median bimodal benefit (here: difference in SC with bimodal fitting compared to unilateral HA fitting for FT at 65 dB SPL) was 10% over the total study period. For a median of 79% of the test subjects, the bimodal benefit was found over the entire period of 36 months.

**Conclusion:**

Over time, SC improves significantly with a CI for the bimodal test subjects. The investigated influencing factors (age, duration of deafness, and degree of hearing loss in the contralateral ear) support the indication for bimodal provision in accordance with the guideline in Germany for cochlear implantation—regardless of age, duration of deafness, and hearing ability of the contralateral ear.

Eine Hörschädigung kann in sozialer Isolation und einer Minderung der Lebensqualität (LQ) resultieren [5, 6]. Dank der (Weiter‑)Entwicklung von Cochleaimplantaten (CI) kann Menschen mit hochgradiger Schwerhörigkeit, die nicht von Hörgeräten (HG) profitieren können, geholfen werden. In der folgenden Längsschnittstudie wurde das Sprachverstehen (SV) von bimodal versorgten CI-Träger:innen im postoperativen Verlauf betrachtet sowie mögliche Einflussfaktoren untersucht.

Die bimodale Hörversorgung einseitig ertaubter Patient:innen mit einem CI bei bestehender kontralateraler HG-Versorgung des besseren Ohrs ist bezüglich des SV und des Richtungshörens in Ruhe sowie im Störgeräusch einer monauralen Versorgung mit HG überlegen [12, 20, 21]. In ruhigen Umgebungen erzielen einseitig Ertaubte oder anderweitig deutlich asymmetrisch Hörende in der Regel ein gutes SV, jedoch wird dieses in alltäglichen Situationen durch Störgeräusche beeinträchtigt [1]. Die bimodale Versorgung stellt einen anspruchsvollen Prozess der zentralen Integration von elektrischer und akustischer Stimulation dar [10]. Die neuronale Plastizität ist aufgrund der Adaption des Gehirns an die neuen Höreindrücke von wesentlicher Bedeutung in der Hörrehabilitation mit CI [25].

Es besteht jedoch Uneinigkeit darüber, inwieweit ein bimodaler Nutzen bei der Versorgung mit HG und CI vorhanden ist. Auf der einen Seite gibt es Untersuchungen, die zeigen, dass bimodal versorgte Patient:innen von dieser Versorgungsart profitieren. Hoppe et al. fanden heraus, dass auch Personen, die auf der HG-versorgten Seite besser hören, einen Vorteil von dem zusätzlichen elektrischen Signal erfahren [12]. Balkenhol et al. verglichen das SV in Ruhe und im Störgeräusch monaural mit dem CI allein mit dem Ergebnis bimodal mit CI und HG nach drei und sechs Monaten nach CI-Implantation. Nach drei Monaten waren die bimodalen Ergebnisse für alle Sprachtests und räumlichen Konditionen signifikant besser im Vergleich zur monauralen CI-Versorgung. Nach sechs Monaten waren die Vergleiche für die Sprachtests mit der bimodalen Versorgung ebenfalls signifikant besser, im Störgeräusch jedoch nicht in allen räumlichen Konditionen [3]. Schon beim Vergleich dieser beiden Publikationen wird deutlich, dass die Betrachtung des bimodalen Nutzens unterschiedlich untersucht wird. Andere Publikationen wie beispielsweise die von Williges et al. kamen zu der Erkenntnis, dass für die bimodale Studiengruppe kein binauraler Summationseffekt nachgewiesen werden konnte und im Wesentlichen immer mit dem besseren Ohr gehört wird [32]. Bei Mok et al. waren die Ergebnisse sehr unterschiedlich: Von den 14 getesteten Teilnehmer:innen zeigten sechs einen signifikanten bimodalen Vorteil bei der Messung der Sprachwahrnehmung bei offenen Sätzen und fünf einen Vorteil bei eng gesetzten Zweisilbern. Allerdings zeigten zwei Teilnehmer:innen in mindestens einem der Sprachwahrnehmungstests eine schlechtere Sprachwahrnehmung bimodal versorgt als mit CI allein [18]. Alle genannten Arbeiten unterscheiden sich hinsichtlich einem oder mehrerer wichtiger Merkmale wie der Gruppengröße, der eingesetzten Sprachtests, den Vertäubungsmethoden und Stimuluskonfigurationen. Darüber hinaus ist die Entwicklung des SV in Ruhe und im Störgeräusch im zeitlichen Verlauf von bimodalen CI-Träger:innen bisher nicht hinreichend untersucht. Allgemein ist bekannt, dass eine Vielzahl von internen und externen Faktoren, wie beispielsweise die Ertaubungsdauer oder die Genese der Schwerhörigkeit, das Ergebnis nach einer Cochleaimplantation beeinflusst [19].

Diese Studie soll anhand einer mittleren Stichprobengröße die langfristige Entwicklung des SV (sowohl in Ruhe als auch in Störgeräusch) bei einer Gruppe bimodaler CI-Träger:innen eingehender analysieren. Besonderes Augenmerk liegt dabei auf dem bimodalen Nutzen und der Untersuchung potenzieller Einflussfaktoren, die sich auf das Behandlungsergebnis der CI-Nutzer:innen auswirken könnten.

## Methodik

In die retrospektive Längsschnittstudie im Paneldesign wurden 54 bimodal versorgte CI-Träger:innen eingeschlossen, die im Zeitraum von 2014 bis 2019 am Universitätsklinikum Tübingen mit einem CI versorgt wurden und mindestens 36 Monate Hörerfahrung mit CI aufwiesen. Sie besuchten während eines Zeitraums von typischerweise zwei bis drei Jahren eine CI-Intervallrehabilitation. Neben regelmäßigen technischen Anpassungen und ärztlichen Untersuchungen erhielten die Rehabilitand:innen hier logopädische, musiktherapeutische und mototherapeutische Angebote in Gruppen- sowie Einzelsettings und psychologische Beratungen und Kommunikationsrunden zum Austausch mit anderen Betroffenen [2].

Die Patient:innen waren zum Untersuchungszeitpunkt mindestens 18 Jahre alt und beherrschten die deutsche Lautsprache fließend. Es wurden die in der Routine erhobenen audiometrischen Daten sowie personenbezogene Daten in die Analysen einbezogen. Um die Bedingungen des bimodalen Hörens zu erfüllen, wurden ausschließlich Studienteilnehmer:innen in die Untersuchung einbezogen, die mit ihrem HG im Freifeld ein SV von mindestens 20 % bei 80 Dezibel (dB) „sound pressure level“ (SPL) im Freiburger Einsilbertest (FBE) aufwiesen.

### Audiometrische Daten

Audiometrische Daten wurden mit einem Audiometer Auritec AT900 oder AT1000 (Fa. Auritec Medizindiagnostische Systeme GmbH, Hamburg, Deutschland) erhoben. Reintonschwellen werden hier als 4‑Frequenz-Mittelwert (0,5, 1, 2, 4 kHz) angegeben und als PTA‑4 („pure-tone average“) bezeichnet. Das SV wurde mit dem FBE [9] (65 und 80 dB SPL) im Freifeld gemessen. Gezeigt wird das SV des CI-versorgten Ohrs bei gleichzeitiger Verdeckung des unversorgten, kontralateralen Ohrs mit 80 dB SPL Breitbandrauschen. Die 50%-Sprachverständnisschwelle (SVS_50_) mit dem Oldenburger Satztest [28] (OlSa, Matrix-Sprachtest, offene Methode), Sprachstimulus im Freifeld von vorn (S_0_). Die SVS_50_ im Störgeräusch wurde mit dem OlSa mit Störgeräusch (Pegel des sprachsimulierenden Rauschens „olnoise“ konstant bei 65 dB SPL, Sprachpegel adaptiv) mit Sprachstimulus von vorn und Störgeräusch von vorn (S_0_N_0_), Störgeräusch von der HG-versorgten Seite (S_0_N_HG_) und Störgeräusch von der CI-versorgten Seite (S_0_N_CI_) evaluiert und als Rauschabstand (SNR, „signal-to-noise ratio“) angegeben. Beim OlSa wurden zwei Trainingslisten durchgeführt, um den Gewöhnungs- und Trainingseffekt zu minimieren [29]. Die eingeschlossenen Versuchspersonen nutzten während der Hörtests mit CI und HG ihr Alltagsprogramm mit den individuell eingestellten Parametern und Automatiken.

### Statistische Auswertung

Alle erhobenen Daten wurden mittels Microsoft-Excel 2022 und der Programmiersprache R [22] in der Version R 4.3.1 mit der Software RStudio 2023.06.2+561 ausgewertet und visuell aufgearbeitet. Das Signifikanzniveau von kleiner als 5 % wird in den weiteren Ausführungen als signifikant, ein Niveau von kleiner als 1 % als sehr signifikant und ein Niveau von kleiner als 0,1 % als hoch signifikant bezeichnet.

Die Prüfung auf Normalverteilung erfolgte mittels Shapiro-Wilk-Test, die Daten waren nicht normalverteilt und es wurden nichtparametrische Tests durchgeführt. Um die Veränderung im zeitlichen Verlauf zu evaluieren, wurde der Friedman-Test für abhängige Stichproben durchgeführt. Bei signifikanten Ergebnissen wurde außerdem eine Post-hoc-Rechnung (Conover-Test) durchgeführt. Um mögliche Einflussfaktoren zu untersuchen, kam der Kruskal-Wallis-Test (mit anschließender Post-hoc-Rechnung – Wilcoxon-Test mit Bonferroni-Korrektur) zum Einsatz. Für die Untersuchung des Zusammenhangs zwischen der Ertaubungsdauer und dem Sprachverstehen wurde die Spearman-Korrelation angewendet.

## Ergebnisse

### Personenbezogene Daten

Die Tab. 1 zeigt die Geschlechts- und Altersverteilung der Studienteilnehmer:innen zum Zeitpunkt der CI-Versorgung (26–82 Jahre), die Fallzahlen gruppiert nach der CI-Seite sowie der Schwerhörigkeit des kontralateral mit HG versorgten Ohrs. Im Mittelwert beträgt das Alter der Studienteilnehmer:innen 63,7 Jahre mit einer Standardabweichung von 13,4 Jahren. Die Eingruppierung der Schwerhörigkeit des HG-Ohrs erfolgte gemäß der WHO-Klassifizierung [17]. Die meisten Studienteilnehmer:innen (56 %) haben einen mittelschweren oder schweren Hörverlust auf dem mit HG versorgten Ohr. Die Ertaubungsdauer des cochleaimplantierten Ohrs wurde anamnestisch erfasst und liegt zwischen 5 Monaten und 62 Jahren. Im Mittel waren die Studienteilnehmer:innen zum Zeitpunkt der CI-Versorgung 15,8 Jahre mit einer Standardabweichung von 17,5 Jahren ertaubt. Da bei einigen Patient:innen anamnestisch kein genauer Ertaubungszeitpunkt evaluiert werden konnte (schwerhörig seit Kindheit/Jugend etc.), wurden für weitere Analysen Gruppen gebildet, die Verteilung kann ebenfalls in Tab. 1 eingesehen werden.

**Tab. 1:** Personenbezogene Daten der Studienteilnehmer:innen, die Stichprobenanzahl ist jeweils als absolute Häufigkeit und als prozentualer Anteil angegeben. Der mittlere Luftleitungshörverlust bei 0,5, 1, 2 und 4 kHz wird als PTA‑4 bezeichnet

**Fallzahlen gruppiert nach Geschlecht**		
***Geschlecht***	***Häufigkeit***	
	*Anzahl n*	*Anteil (%)*
Weiblich	19	35
Männlich	35	65
**Fallzahlen gruppiert nach Alter bei Versorgung**		
***Alter (Jahre)***	***Häufigkeit***	
	*Anzahl n*	*Anteil (%)*
Bis 50 Jahre	7	13
51 bis 60 Jahre	13	24
61 bis 70 Jahre	13	24
Über 70 Jahre	21	39
**Fallzahlen gruppiert nach CI-Seite**		
***CI-Seite***	***Häufigkeit***	
	*Anzahl n*	*Anteil (%)*
Rechts	30	56
Links	24	44
**Fallzahlen gruppiert nach Dauer der Taubheit (anamnestisch)**		
***Dauer der Taubheit ipsilateral (Jahre)***	***Häufigkeit***	
	*Anzahl n*	*Anteil (%)*
< 5 Jahre	10	19
5–9 Jahre	9	17
10–19 Jahre	11	20
20–29 Jahre	6	11
> 30 Jahre	18	33
**Fallzahlen gruppiert nach Schwerhörigkeit kontralaterales Ohr**		
***PTA‑4***	***Häufigkeit***	
	*Anzahl n*	*Anteil (%)*
20 bis < 35 dB: leichter HV	5	9
35 bis < 50 dB: mäßiger HV	10	19
50 bis < 65 dB: mittelschwerer HV	17	31
65 bis < 80 dB: schwerer HV	13	24
80 bis < 95 dB: hochgradiger HV	8	15
95 dB oder mehr: vollständiger HV	1	2

**Abb. 1:**
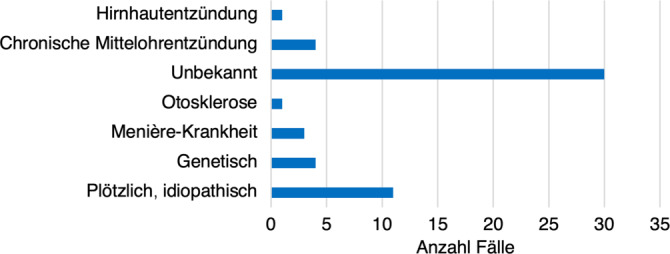
Ertaubungsursachen nach Blamey, abgedruckt sind lediglich die vorkommenden Ursachen, folgende Ursachen kamen in der hier untersuchten Versuchspersonengruppe nicht vor: akustisches Trauma, Sonstiges, Ototoxizität, Labyrinthitis, Fraktur des Schläfenbeins, Akustikusneurinom, Auditorische-Neuropathie-Spektrum-Störung

Die Ertaubungsursachen der Studienteilnehmer:innen können Abb. 1 entnommen werden. Es wurden die Kategorien nach Blamey herangezogen, in der Abbildung sind lediglich die vorkommenden Kategorien abgebildet [4].

Die Tab. 2 zeigt zur Übersicht die Daten des CI-versorgten Ohrs wie Implantathersteller, die jeweils eingesetzten Implantat- und Elektrodentypen sowie die verwendeten Audioprozessoren. Es kamen die gängigen Kodierungsstrategien der Hersteller zum Einsatz.

**Tab. 2:** Übersicht der herstellerspezifischen Eigenschaften der Studienteilnehmer:innen

Hersteller	Fälle	Implantat		Elektroden		Audioprozessor	
*MED-EL*	38	35	Synchrony	34	Flex-28-Elektrode	2	Opus 2
						3	Rondo
		3	Synchrony 2	4	Flex-Soft-Elektrode	30	Sonnet
						3	Rondo 2
*Cochlear*	8	8	CI 522	8	Slim-Straight-Elektrode	4	N6
						2	Kanso
						2	N7
*AB*	5	4	HiRes Ultra CI	5	HiFocus-MS-Elektrode	5	Naida CI Q 90
		1	HiRes 90 K Advantage CI				
*Oticon Medical*	3	2	Neuro Zti EVO	3	EVO-Elektrode	2	Neuro 2
		1	DIGISONIC SP EVO			1	Saphyr neo

#### Audiometrische Daten

**Abb. 2:**
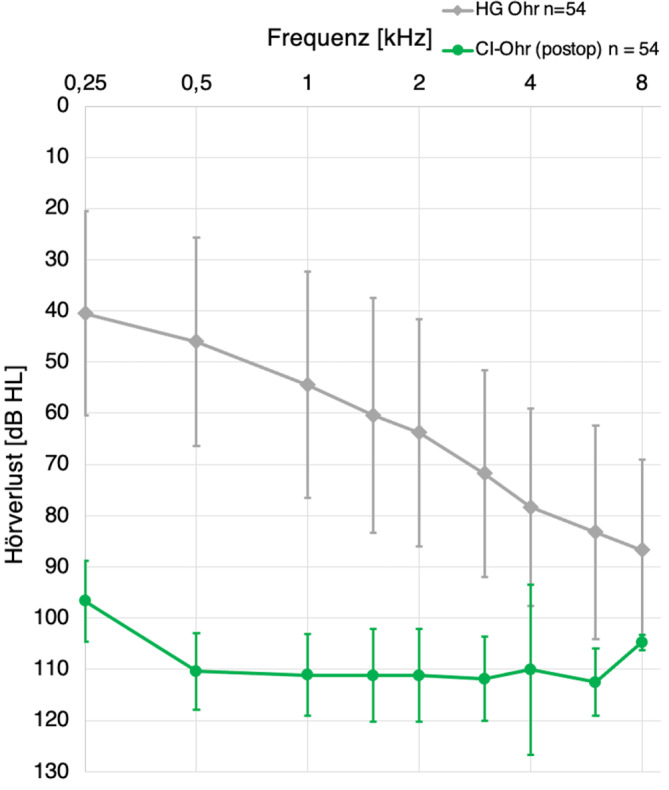
Gemitteltes Tonaudiogramm ± Standardabweichung des mit HG versorgten Ohrs in *Grau* und des mit CI versorgten Ohrs in *Grün*. Bei Erreichen der Audiometerleistungsgrenze wurde der jeweilige Grenzwert addiert mit 5 dB HL als Schwelle angegeben

Die Abb. 2 zeigt das gemittelte Tonaudiogramm ± Standardabweichung für jede Messfrequenz des mit HG versorgten Ohrs und das gemittelte postoperative Tonaudiogramm des CI-versorgten Ohrs. In Fällen, in denen die Audiometerleistungsgrenze erreicht wurde, wurde der jeweilige Grenzwert plus 5 dB HL als Schwelle angegeben. Der mittlere PTA-4-Wert beträgt für das mit HG versorgte Ohr 60,65 ± 13,85 dB HL und für das CI-Ohr 89,65 ± 7,75 dB HL.

**Abb. 3:**
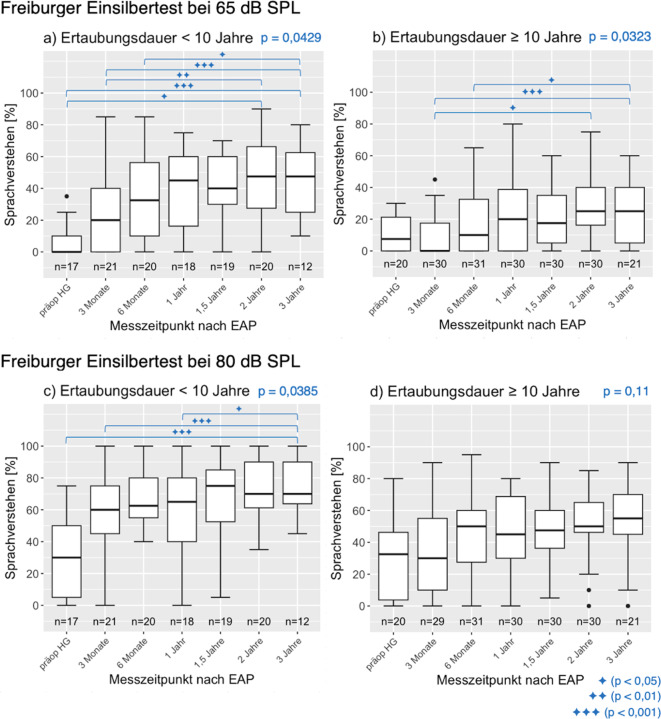
Median, Interquartilsbereich und Wertebereich des Sprachverstehens des CI-versorgten Ohrs in Abhängigkeit vom Messzeitpunkt nach der Erstanpassung (falls der Wertebereich mehr als das 1,5-Fache des Interquartilsabstands betrug, wird dieser angezeigt und darüberhinausgehende Werte als *Punkte* markiert – hier und in folgenden Abbildungen). Präoperativ ist der mit HG versorgte Wert angegeben, wenn die Versuchsperson ein HG getragen hat. *Linke Spalte* Ertaubungsdauer < 10 Jahre (**a, c**). *Rechte Spalte *Ertaubungsdauer ≥ 10 Jahre (**b, d**). Die Klammern und Symbole zeigen, zwischen welchen Messzeitpunkten ein signifikanter Unterschied nachgewiesen werden konnte

Zur Analyse des FBE-Tests wurde das SV für die Pegel 65 dB SPL und 80 dB SPL für die CI-versorgte Seite getrennt in zwei Gruppen (Ertaubungsdauer < und ≥ 10 Jahre) im Verlauf verglichen. Die Ergebnisse sind anhand von Box-Whisker-Plots dargestellt und können Abb. 3 entnommen werden.

Der Friedman-Test zeigte eine signifikante Verbesserung des SV im zeitlichen Verlauf für die Ertaubungsgruppe < 10 Jahre, sowohl für 65 dB SPL als auch für 80 dB SPL. Hingegen war in der Ertaubungsgruppe ≥ 10 Jahre nur eine signifikante Verbesserung für 65 dB SPL nachweisbar. Aus Abb. 3 ist ersichtlich, dass der Paarvergleich präoperativ mit HG versus drei Monate postoperativ mit dem CI das Signifikanzkriterium für beide Ertaubungsgruppen und beide Sprachschallpegel verfehlte.

Für die Gruppe Ertaubungsdauer < 10 Jahre ergab sich nach drei Jahren für 65 dB SPL im Median ein SV von 47,5 % (25 % für Gruppe Ertaubungsdauer ≥ 10 Jahre) und für 80 dB SPL ein SV von 70 % (55 % für Gruppe Ertaubungsdauer ≥ 10 Jahre). Zwischen welchen Messzeitpunkten die Verbesserung signifikant ist, ist anhand der Klammern in Abb. 3 ersichtlich.

Um größere Fallzahlen miteinander vergleichen zu können und eine allgemeinere Aussage der Veränderung über die Zeit treffen zu können, wurden die Daten des FBE zusammengefasst ausgewertet. Es wurden alle Werte bis zu einem Jahr und alle Werte über einem Jahr zusammengefasst. Sowohl für 65 dB SPL als auch für 80 dB SPL waren die erreichten Werte im FBE für die gesamte Versuchspersonengruppe zwischen präoperativ und dem Wert nach einem Jahr, zwischen präoperativ und dem Wert nach zwei Jahren und zwischen dem Wert nach einem Jahr und dem Wert nach zwei Jahren signifikant besser (Tab. 3).

**Tab. 3:** Gruppierte Daten des Freiburger Einsilbertests für die gesamte Versuchspersonengruppe, aufgeteilt bis zu einem Jahr und über einem Jahr. Präoperativ ist die Fallzahl deutlich geringer, da nicht alle Versuchspersonen vor der Implantation ein Hörgerät nutzten

FBE % korrekt		*65* *dB SPL: p* = 0,00003		*80* *dB SPL: p* = 0,0008	
	*Anzahl*	*Median*	*75* *%-Quartil*	*Median*	*75* *%-Quartil*
*Präop.*	37	0	20	30	50
*Bis 1 Jahr*	150	20	40	50	70
*Über 1 Jahr*	132	30	50	60	80

Um den Einfluss des Alters bei der Versorgung auf das Sprachverstehen mit CI zu untersuchen, wurden vier Altersgruppen gebildet und der beste Wert des SV innerhalb der 36 Monate für die Analyse ausgewählt. Es zeigte sich (nach Post-hoc-Rechnung) kein signifikanter Unterschied in den verschiedenen Altersgruppen beim FBE (Tab. 4).

**Tab. 4:** Sprachverstehen in % (Median, *MW* Mittelwert, *IQA* Interquartilsabstand und *SD* Standardabweichung) im Freiburger Einsilbertest für 65 dB SPL und 80 dB SPL. Im Post-hoc-Test waren die Paarvergleiche nicht signifikant

Altersgruppen		*FBE* *% korrekt* – 65 dB SPL *p* = 0,0402				*FBE* *% korrekt* – 80 dB SPL *p* = 0,0841			
	*Anzahl*	*Median*	*IQA*	*MW*	*SD*	*Median*	*IQA*	*MW*	*SD*
*Bis 50 Jahre*	7	55	17,5	55	15,28	80	12,5	77,14	14,1
*51 bis 60 Jahre*	13	70	35	62,69	24,72	85	30	81,92	19,1
*61 bis 70 Jahre*	13	40	45	41,92	23,23	75	30	72,69	19,75
*Über 70 Jahre*	21	45	35	40	21,91	70	20	65,24	17,71

Der Grad der Schwerhörigkeit des mit HG versorgten Ohrs wurde als weiterer möglicher Einflussfaktor für das SV des CI-Ohrs untersucht. Da die Gruppengrößen sehr unterschiedlich waren, wurde der hochgradige und der vollständige Hörverlust zu einer Gruppe zusammengefasst. Für den FBE ergab sich sowohl für 65 dB SPL als auch für 80 dB SPL kein signifikanter Unterschied zwischen den Gruppen des Grads der Schwerhörigkeit des HG-Ohrs.

**Abb. 4:**
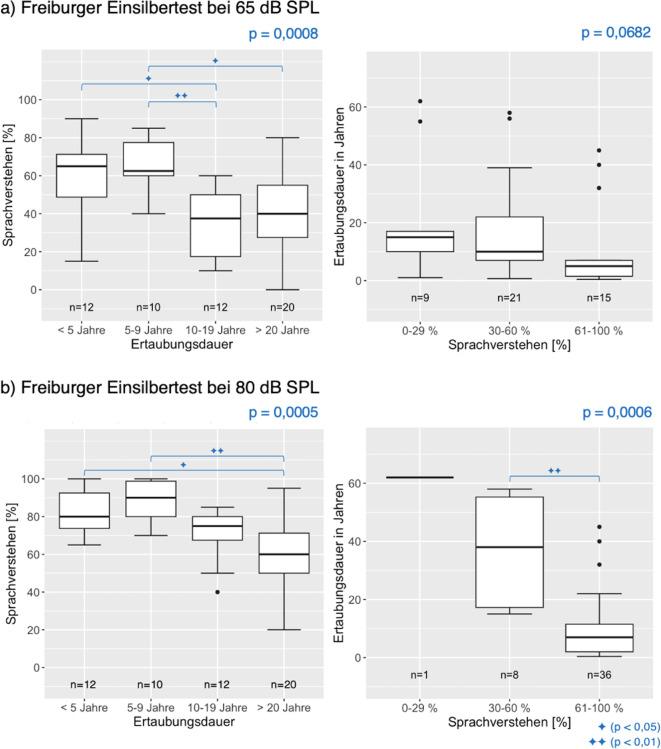
*Linke Spalte *Median, Interquartilsbereich und Wertebereich des besten Sprachverstehens im Freiburger Einsilbertest innerhalb von 36 Monaten in Abhängigkeit von der Ertaubungsdauer; *rechte Spalte *Ertaubungsdauer in Abhängigkeit vom in drei Gruppen aufgeteilten Sprachverständnis bei 65 dB SPL (**a**) und 80 dB SPL (**b**). Zwischen welchen Messzeitpunkten ein signifikanter Unterschied nachgewiesen werden konnte, ist anhand der Klammern und Symbole ersichtlich. Man beachte, dass die Gruppeneinteilung der Ertaubungsdauer in der *linken Spalte* ermöglicht, Patient:innen einzubeziehen, für die die Ertaubungsdauer nicht genau angegeben werden konnte (z. B. „seit Kindheit“); dies begründet die unterschiedliche Summe der Fallzahlen

Das maximal erreichte SV im FBE wurde außerdem für die Analyse des Einflusses der Ertaubungsdauer genutzt. Die Proband:innen wurden abhängig von ihrer Ertaubungsdauer in vier Gruppen eingeteilt. Die Ergebnisse sind im linken Teil von Abb. 4 dargestellt. Sowohl für 65 dB SPL als auch für 80 dB SPL war ein hoch signifikanter Unterschied nachweisbar, wobei eine längere Ertaubungszeit zu einem schlechteren Ergebnis im FBE führte. Für eine weitere Analyse wurde das beste Ergebnis des FBE in drei Performancegruppen eingeteilt (Sprachverstehen 0–29 %, 30–60 % und 61–100 %) und in Abhängigkeit der anamnestisch erhobenen Ertaubungsdauer in Jahren untersucht. Proband:innen, die den Zeitpunkt der Ertaubung nicht sicher benennen konnten, mussten für diese Analyse ausgeschlossen werden. Die Stichprobenanzahl sowie die Verteilung können den Box-Whisker-Plots der rechten Spalte in Abb. 4 entnommen werden. Besonders eindeutig zeigt sich die Abhängigkeit der Ertaubungsdauer bei 80 dB SPL mit einem sehr signifikanten Unterschied zwischen den mittleren und den sehr guten Performern.

**Abb. 5:**
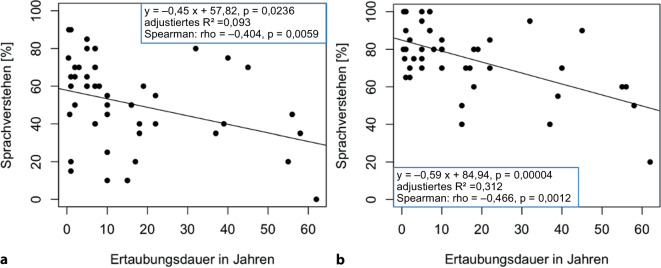
Korrelation der Ertaubungsdauer mit dem Sprachverstehen (**a** FBE bei 65 dB SPL,** b** FBE bei 80 dB SPL). Dargestellt sind die einzelnen *Datenpunkte* und die Regressionsgerade sowie die Kennzahlen der Korrelationsrechnung (*n* = 45)

In Abb. 5 wurde auf der x‑Achse die Ertaubungsdauer in Jahren und auf der y‑Achse das maximal erreichte Messergebnis beim FBE bei 65 dB SPL (Abb. 5a) und 80 dB SPL (Abb. 5b) aufgetragen. Es zeigt sich hier eine negative Korrelation. Die Spearman-Korrelation ergibt für 65 dB SPL ein rho von −0,404 (*p* = 0,0059) und für 80 dB SPL ein rho von −0,466 (*p* = 0,0012).

**Abb. 6:**
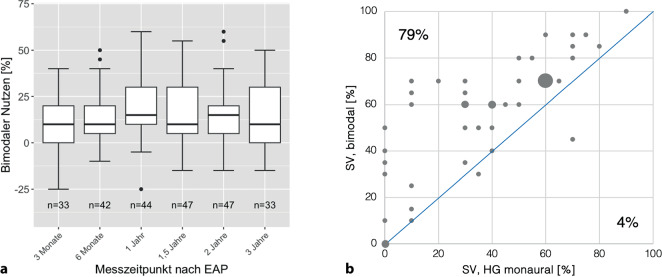
Median, Interquartilsbereich und Wertebereich des Sprachverstehens für den bimodalen Nutzen des Freiburger Einsilbertests im Freifeld (Differenz aus dem Sprachverstehen mit bimodaler Versorgung gegenüber einseitiger HG-Versorgung) bei 65 dB SPL **a** für die einzelnen Messzeitpunkte. **b** Verteilung des bimodalen Nutzens für 1 Jahr postoperativ. Für 79 % der Patient:innen ist der bimodale Nutzen vorhanden. Der *Durchmesser der Punkte* ist proportional zur Häufigkeit der Fallzahl

Die Abb. 6 zeigt den bimodalen Nutzen der CI-Versorgung. Dieser wurde hier definiert als die Differenz aus dem SV in Prozent bei bimodaler Versorgung gegenüber dem SV mit einseitiger Versorgung mit HG (FBE, 65 dB SPL). Ist das Ergebnis positiv, ist die bimodale Versorgung der einseitigen HG-Versorgung überlegen. Im Median betrug der bimodale Nutzen beim FBE über die gesamte Untersuchungszeit für 65 dB SPL 10 %, im Mittel 15 %. Der Nutzen zeigt keinen systematischen Zeitverlauf (Abb. 6a). Die Abb. 6b zeigt die Verteilung des bimodalen Nutzens für den Zeitpunkt ein Jahr postoperativ. Diese Verteilung zeigte ebenfalls keinen ausgeprägten Zeitverlauf im Untersuchungszeitraum: Gemittelt über den gesamten Zeitverlauf von 36 Monaten war der bimodale Nutzen im Mittel bei 78 ± 5,0 % (Median: 79 %) der Versuchspersonen nachweisbar. Die Abb. 6a zeigt anhand des unteren Whiskers, dass es auch vereinzelt Versuchspersonen gab, bei denen kein bimodaler Nutzen nachgewiesen werden konnte (Verschlechterung bis maximal 25 % mit der bimodalen Versorgung gegenüber der einseitigen HG-Versorgung).

Zusätzlich wurde der bimodale Nutzen in Ruhe als ein bereits von Sheffield et al. verwendetes relatives Maß definiert [26]. Dies wird als normierte bimodale Summierung bezeichnet. Es wurde als der Prozentsatz des möglichen Nutzens berechnet, der in Bezug auf das HG erzielt wurde ([SV bimodal – SV HG]/[100 – SV HG] × 100). Zum Zeitpunkt ein Jahr postoperativ war die normierte bimodale Summierung im Mittel 31,9 ± 29,4 % (Median: 31,7 %).

**Abb. 7:**
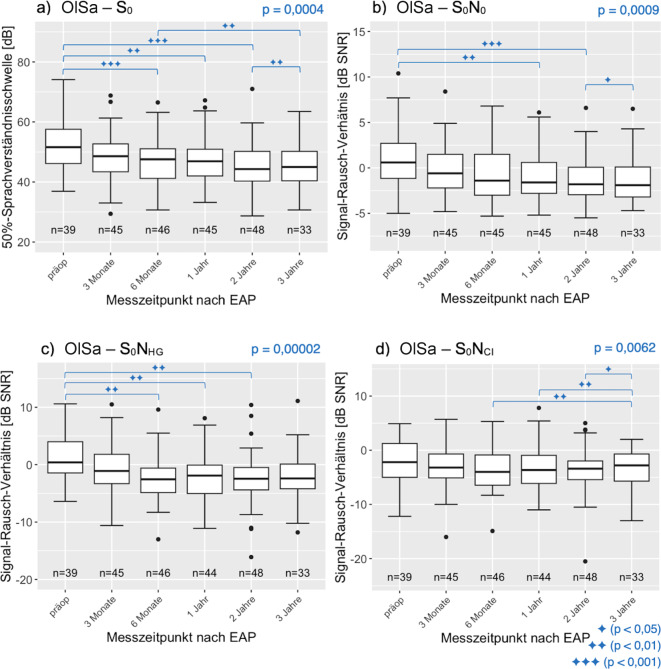
Median, Interquartilsbereich und Wertebereich der 50%-Sprachverständnisschwelle in Ruhe (**a**) bzw. Rauschabstand im Störgeräusch beim OlSa in Abhängigkeit vom Messzeitpunkt nach der Erstanpassung (*EAP*, **b**, **c**, **d**). Die Messungen wurden „best aided“ durchgeführt. Die Klammern und Symbole zeigen, zwischen welchen Messzeitpunkten ein signifikanter Unterschied nachgewiesen werden konnte

Beim OlSa wurden die einzelnen Testkonfigurationen (S_0_, S_0_N_0_, S_0_N_HG_, S_0_N_CI_) ausgewertet (Abb. 7). Der Friedman-Test ergab über den gesamten Datensatz für die Konfigurationen S_0_, S_0_N_0_ und S_0_N_HG_ eine hoch signifikante und für S_0_N_CI_ eine sehr signifikante Veränderung. Zwischen welchen Messzeitpunkten die Verbesserung signifikant ist, ist anhand der Klammern ersichtlich.

Die Stichprobenzahl der OlSa-Messungen ist geringer als die des FBE, da in 5 % der Messungen der OlSa abgebrochen werden musste, beispielsweise bei Erreichen der Unbehaglichkeitsschwelle oder der Audiometerleistungsgrenze.

**Abb. 8:**
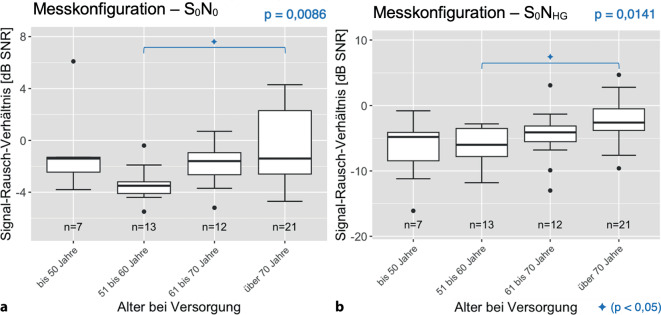
Median, Interquartilsbereich und Wertebereich des Rauschabstands für die jeweiligen Altersgruppen beim OlSa im Störgeräusch. Die Klammern und Symbole (**a** Konfiguration S_0_N_0_, **b** Konfiguration S_0_N_HG_) zeigen, zwischen welchen Gruppen ein signifikanter Unterschied nachgewiesen werden konnte. Die Gruppe mit dem Versorgungsalter über 70 Jahre benötigt einen signifikant höheren SNR im Vergleich zu jüngeren Altersgruppen (51 bis 60 Jahre)

Die Auswertung in den Altersgruppen ergab für die Messkonfiguration S_0_ und S_0_N_CI_ keinen signifikanten Unterschied (nach Post-hoc-Rechnung). Die Konfiguration S_0_N_0_ und S_0_N_HG_ ergab einen sehr signifikanten bzw. signifikanten Unterschied (Abb. 8).

## Diskussion

Im Rahmen dieser Studie wurde das SV bei bimodaler Versorgung über einen längeren Zeitraum von 36 Monaten retrospektiv untersucht und der bimodale Nutzen unter klinischen Gesichtspunkten näher beleuchtet. Im Fokus stand die klinische Frage, inwieweit bei bestehender HG-Versorgung ein zusätzliches CI für die Patient:innen einen Mehrwert bringt. Im Median verbesserte sich das Ergebnis bei bimodaler Versorgung der Patientengruppe, die auf der HG-versorgten Seite einen PTA‑4 von 61 dB HL aufwies, über den gesamten Untersuchungszeitraum für den FBE-Test in Ruhe bei 65 dB SPL um zehn Prozentpunkte. Das ist ein nicht unbedeutender Zugewinn. Eine Verbesserung war im Median bei 79 % der Studienteilnehmer:innen nachweisbar. Die Abb. 6b zeigt, dass es für den Messzeitpunkt nach zwölf Monaten zwei Patient:innen gab, für die formal ein negativer bimodaler Nutzen errechnet wurde. Eine:r davon zeigte mit der bimodalen Versorgung eine erhebliche Verringerung des SV von 25 %, der zweite von 5 % (was einem Wort der getesteten Liste des FBE entspricht). Schaut man sich den bimodalen Nutzen über alle fünf postoperativen Messzeitpunkte an, so beträgt dieser für die erste Versuchsperson mit formal negativem bimodalen Nutzen im Median –5 % (Mittelwert: –7 %). Diese Versuchsperson scheint also auch gemittelt im FBE einen negativen bimodalen Nutzen aufzuweisen, die 25 % im Test nach einem Jahr stellen aber eine Ausnahme innerhalb der 36 Monate dar. Der ermittelte Rauschabstand der SVS_50_ im OlSa in der Messkonfiguration S_0_N_HG_ lag für diese Versuchsperson präoperativ bei +2,9 dB SNR (S_0_N_0_: +0,2 dB SNR), und zwölf Monate postoperativ wurde ein Rauschabstand von +0,3 dB SNR erreicht (S_0_N_0_: −2,3 dB SNR). Zum Vergleich: Nach zwölf Monaten wird in der Konfiguration S_0_N_CI_ ein Rauschabstand von −5,8 dB SNR erzielt. Es zeigt sich also auch bei diesem Fall ein audiologischer Nutzen nach bimodaler Versorgung, auch wenn die o. g. Werte zeigen, dass diese Person vom HG sehr viel mehr profitiert. Es sollte außerdem bedacht werden, dass es für Patient:innen, welche mit dem HG allein bereits ein sehr gutes SV erzielen, deutlich schwieriger ist einen positiven bimodalen Nutzen im FBE nachzuweisen. Bei der Betrachtung von Einzelfällen muss darüber hinaus immer die inhärente Schwankung der eingesetzten Messverfahren berücksichtigt werden [33].

Beim Satztest im Rauschen (OlSa) wurde bimodal unter S_0_N_0_ ein SNR von −2 dB erreicht, hingegen bei S_0_N_HG_ ein SNR von −3 dB. Hier erscheint der Zugewinn von 1 dB auf den ersten Blick gering. Allerdings ist zu erwarten, dass in dieser Messbedingung das HG nichts oder fast nichts zum SV im Störgeräusch beitragen kann, weil aufgrund der Verschiebung des Störgeräuschs auf HG-Seite ein Pegelzuwachs in der Größenordnung von 3–4 dB erfolgt und daher mit einer Steigung der psychometrischen Kurve von ca. 12 %/dB [32] das Wortverstehen um 36–48 % vom 50 %-Wert abgesunken sein sollte. Unter dieser Annahme kann man das SNR für die Messbedingung CI allein bei S_0_N_0_ mit 0 dB abschätzen (−2 dB +2 dB Vorteil durch beidohriges Hören). Wenn diese Abschätzung stimmt, dann war der Vorteil durch die CI-Implantation bei S_0_N_0_ effektiv 3 dB. Die Abschätzung steht unter dem vergröberten Vorbehalt, dass mit dem Median gerechnet wird und einbezogen wird, dass der Median für S_0_N_HG_ und S_0_N_CI_ fast identisch war.

Im Vergleich mit der Literatur muss einbezogen werden, dass sich in der Regel mindestens einer der wesentlichen Faktoren wie eingesetzte Sprachtests, Anregungsbedingungen, Altersverteilung, Hörstatus, Ertaubungsdauer unterscheiden. Weißgerber et al. [31] haben bei einer Gruppe von 19 bimodal versorgten CI-Träger:innen das SV im modulierten Störgeräusch mit dem SV einer bilateral mit HG versorgten Gruppe und zwei normalhörenden Gruppen mit unterschiedlicher Altersstruktur verglichen. Dabei wurde zusätzlich auch das Einsilberverstehen des FBE erfasst. Es resultierte ein mittleres Einsilberverstehen auf dem mit CI versorgten Ohr von 77,9 ± 15,6 %. Für die hier untersuchte Gruppe ergab sich für die Gruppe Ertaubungsdauer < 10 Jahre nach drei Jahren im Median ein SV von 47,5 %. Das ist eine deutliche Abweichung, zumal die demografischen Daten wie das Alter bei Versorgung vergleichbar sind (Durchschnittsalter: bei Weißgerber 70,7 ± 6,2 Jahre; untersuchte Studiengruppe 63,7 ± 13,4 Jahre). Der PTA des mit HG versorgten Ohrs ist ebenfalls in einem ähnlichen Bereich (mittlerer PTA: bei Weißgerber 70,3 ± 14,2 dB HL; untersuchte Studiengruppe 60,65 ± 13,85 dB HL). Das deutlich bessere Wortverstehen resultiert vermutlich aus der Vertäubungsmethodik. Bei Weißgerber et al. kam kein Vertäubungsgeräusch zum Einsatz, stattdessen wurde bei der Messung des CI allein das kontralaterale Ohr mit einem Ohrstöpsel und zusätzlich einem ohrumschließenden Kapselgehörschutz isoliert. In der hier untersuchten Studiengruppe wurde das HG-Ohr dagegen über einen Kopfhörer mit 80 dB SPL Breitbandrauschen vertäubt. Vergleicht man die Messungen des OlSa, die mit derselben Konfiguration durchgeführt wurden (Störgeräusch konstant bei 65 dB SPL und Sprache adaptiv), schneidet die in der vorliegenden Studie untersuchte Gruppe geringfügig besser ab (Weißgerber: S_0_N_0_ −1,5 dB SNR; untersuchte Studiengruppe nach drei Jahren: −1,9 dB SNR) [31].

In einer Studie von Balkenhol et al. wurde bei 15 bimodal versorgten CI-Träger:innen der FBE mit einem Sprachschallpegel von 70 dB SPL in Ruhe sowie im Störgeräusch der OlSa (Störgeräusch konstant bei 60 dB SPL und Sprache adaptiv) gemessen. Bei Balkenhol et al. erreichten die CI-Träger:innen nach drei Monaten im FBE bei 70 dB SPL mit dem CI-Ohr ein SV von 36 % und nach sechs Monaten ein SV von 46 %. Für die Ertaubungsgruppe < 10 Jahre lag der FBE nach drei Monaten im Median bei einem SV von 20 % und nach sechs Monaten bei einem SV von 32,5 %, also ebenfalls etwas schlechter als bei Balkenhol et al. Im Störgeräusch erreichten die Patient:innen bei Balkenhol et al. nach drei Monaten mit der bimodalen Versorgung für S_0_N_0_ ein SVS_50_ von +1 dB SNR (S_0_N_CI_: +0,3 dB SNR; S_0_N_HG_: +1 dB SNR) und nach sechs Monaten für S_0_N_0_ ein SVS_50_ von +0,5 dB SNR (S_0_N_CI_: −1 dB SNR; S_0_N_HG_: −0,8 dB SNR). Für die hier untersuchte Gruppe war das SVS_50_ bimodal versorgt nach drei Monaten für S_0_N_0_ im Median −0,6 dB SNR (S_0_N_CI_:−3,2 dB SNR; S_0_N_HG_: −1,1 dB SNR) und nach sechs Monaten für S_0_N_0_ −1,4 dB SNR (S_0_N_CI_: −4 dB SNR; S_0_N_HG_: −2,55 dB SNR). Im Störgeräusch schnitt die in der vorliegenden Studie untersuchte Gruppe damit etwas besser ab. Die Vertäubung im FBE wurde unterschiedlich umgesetzt, bei Balkenhol et al. kamen Einsteckhörer mit einem Vertäubungspegel von 65 dB SPL weißem Rauschen zum Einsatz [3]. Es darf angenommen werden, dass das wesentlich lautere Vertäubungsgeräusch in der vorliegenden Studie aufgrund von zentraler Maskierung das SV mit dem CI deutlich erschwerte.

Das Phänomen der zentralen Maskierung bei kontralateralen Verdeckungsgeräuschen wurde z. B. an einer Gruppe normalhörender Proband:innen untersucht und zeigte Effekte einer Schwellenverschiebung von 1 bis 11 dB [8]. Da die spektrale und zeitliche Auflösung beim Hören mit einem CI im Vergleich zum gesunden Ohr deutlich reduziert ist, ist dieser Effekt möglicherweise ausgeprägter. Die im Vergleich zu anderen Veröffentlichungen guten Ergebnisse im OlSa unterstützen die Annahme des negativen Einflusses der Vertäubung auf den FBE. Williges et al. nutzen 2019 ebenfalls den OlSa und verglichen die Ergebnisse von bimodalen CI-Träger:innen, SSD-Patienten und einer normalhörenden Vergleichsgruppe. Wie bereits erwähnt, konnte in beiden CI-Gruppen kein binauraler Effekt nachgewiesen werden. Die bimodale Gruppe hatte ein ähnliches Alter (Median: 62,5 Jahre) und ebenfalls eine vergleichbar lange Ertaubungsdauer (Mittelwert: 13,9 Jahre) wie die hier untersuchte Vergleichsgruppe. Die Messkonfiguration im OlSa unterschied sich (Sprache 65 dB SPL konstant, Rauschen adaptiv [„olnoise“]). Die Proband:innen bei Williges erzielten mit S_0_N_0_ = −4 dB SNR, S_0_N_CI_ = −6,5 dB SNR, S_0_N_HG_ = −4,5 dB SNR bessere Ergebnisse als die Versuchspersonen in der hier untersuchten Gruppe: S_0_N_0_ = −2 dB SNR, S_0_N_CI_ = −4 dB SNR, S_0_N_HG_ = −2,5 dB SNR. Zu den wichtigen Unterschieden zählt sicherlich neben der geringen Gruppengröße (bimodale Gruppe: *n* = 8) die virtuelle Schallstimulation: direkte Signalversorgung des CI, Einsteckhörer kontralateral, Hörgerätefunktion sowie räumliche Akustik simuliert. Hiervon sind sowohl förderliche wie hinderliche Einflüsse zu erwarten, beispielsweise wird dadurch jedes zusätzliche Störgeräusch sowie Einfluss von Raumresonanzen ausgeschlossen (förderlich), andererseits auch SNR-Erhöhung durch Kopfbewegungen (hinderlich) [32].

Eine aktuelle Publikation aus dem Jahr 2023 von Rader et al. beschäftigte sich mit der Abhängigkeit der cochleären Abdeckung auf das SV bei einer 39-köpfigen bimodalen Versuchspersonengruppe und nutzte als Sprachtest ebenfalls den FBE in Ruhe bei 65 dB SPL. Das Alter bei Implantation war mit einem Median von 65 Jahren vergleichbar zur hier untersuchten Studiengruppe, die Ertaubungsdauer wurde nicht angegeben ebenso wie der Vertäubungspegel des kontralateral mit HG versorgten Ohrs bei der Durchführung des FBE. Über die gesamte Kohorte verschlechterte sich das mit dem CI versorgte Ohr von 20 % des Gruppenmedians bei der präoperativen Messung mit HG zunächst auf 0 % bei der Erstanpassung und verbesserte sich anschließend stetig. Bei der Ein-Monats-Kontrolle lag das SV bei 25 %, nach drei Monaten bei 30 % und nach einem Jahr bei 40 % [23]. Die Ergebnisse sind vergleichbar mit den Ergebnissen der in dieser Studie untersuchten Versuchspersonengruppe mit einer Ertaubungsdauer < 10 Jahre, die Ertaubungsdauer ≥ 10 Jahre schnitt schlechter ab.

Bei einer sehr großen Studiengruppe von 148 bimodal versorgten CI-Träger:innen untersuchten Hoppe et al. [12] das SV in Ruhe und im Störgeräusch mit dem Göttinger Satztest (GöSa). Das Durchschnittsalter der CI-Träger:innen betrug 60,5 ± 16 Jahre, die Erfahrung mit dem CI im Mittel 28 Monate (6–176Monate). Der GöSa wurde in Ruhe bei 65 dB SPL oder im Störgeräusch in der Konfiguration S_0_N_0_ mit festem Sprachschallpegel von 65 dB SPL und adaptiv gesteuertem Störgeräuschpegel durchgeführt. Die Messung monaural mit CI erfolgte ohne HG, mit Ohrstöpsel im HG-Ohr und Verdeckung mittels Kopfhörer in adäquater Lautstärke (der Pegel wird nicht angegeben). In Ruhe betrug das SV bei 65 dB SPL 86 %, zum Störgeräusch betrug der Rauschabstand für die Konfiguration S_0_N_0_ +7,2 dB SNR [12]. OlSa und GöSa können nicht direkt miteinander verglichen werden, man kann nur über die Abhängigkeit zwischen PTA und SVS_50_ für OlSa und GöSa eine Umrechnung anstellen. Die hier untersuchte Studiengruppe erreichte im OlSa in der Konfiguration S_0_N_0_ einen SVS_50_ von −2 dB SNR, das wird gewöhnlich bei einem PTA von 38 dB HL erreicht [30]. Bei diesem PTA wird für den GöSa ein SVS_50_ von ungefähr −1 dB SNR erwartet [27]. Das bedeutet, die hier untersuchte Studiengruppe müsste im GöSa theoretisch einen SVS_50_ von −1 dB SNR erreichen. Den Rauschabstand von +7,2 dB SNR in der Studie von Hoppe würde man auf ein SNR von +4,2 bis +5,2 dB für binaural symmetrisches Hören umrechnen, bliebe damit aber noch deutlich ungünstiger und nicht leicht erklärbar. Der bimodale Nutzen wurde in der Publikation von Hoppe et al. umfassend untersucht. Bezogen auf das HG war der bimodale Nutzen in 79 % der Fälle gegeben. Dieses Ergebnis ist vergleichbar mit den hier ermittelten Ergebnissen, bei denen im Median ebenfalls für 79 % der Versuchspersonen für den gesamten Zeitraum ein bimodaler Nutzen nachgewiesen werden konnte.

Die genannten Vergleiche zur Literatur machen deutlich, wie wichtig die Berücksichtigung der in den Tests verwendeten Randbedingungen wie Pegel und Hörer bzw. Isolation der Verdeckung oder eben auch der Einstellung der HG bzw. CI für die Vergleichbarkeit ist (hier: Alltagsprogramm inkl. Geräuschunterdrückung; Weißgerber et al.: Alltagsprogramm mit Standardmikrofondirektionalität (Subniere) und Geräuschunterdrückung deaktiviert; bei Hoppe et al. keine Angaben zur Einstellung von HG oder CI). Allerdings wird von einem abgeschalteten Rauschunterdrückungsprogramm bei stationärem Rauschen kein wesentlicher Effekt erwartet, solange keine Kopplung der beiden Hörgeräteelektroniken vorliegt, die den Effekt des „spatial release of masking“ ausnutzen kann.

Die größten Effekte der CI-Versorgung auf die Hörentwicklung treten innerhalb des ersten Jahres nach der Operation auf, was den Box-Whisker-Plots in Abb. 3 entnommen werden kann. In dieser Zeit findet die überwiegende Adaption an die neuen Höreindrücke statt, danach scheint sich der Höreindruck bei vielen Patientinnen und Patienten zu stabilisieren, und die weitere Hörentwicklung verläuft dann kleinschrittiger [4, 7]. Bei der Auswertung der gruppierten Daten für den FBE zeigte sich ein signifikanter Unterschied zwischen präoperativ und dem Wert nach einem Jahr und auch zwischen dem Wert nach einem Jahr und dem Wert nach zwei Jahren für die gesamte Versuchspersonengruppe. Dies unterstreicht die Bedeutung einer Intervallrehabilitation, die CI-Träger:innen über einen längeren Zeitraum begleitet.

Für die verschiedenen Altersgruppen ergab sich bei den Sprachtests in Ruhe (FBE und OlSa S_0_) kein signifikanter Unterschied (nach Post-hoc-Rechnung), was die derzeitige Indikationsstellung ohne Altersobergrenze unterstützen kann. Ähnliche Ergebnisse ergaben sich auch z. B. in Veröffentlichungen von Hinder et al. und Kraaijenga et al. [10, 14]. Für anspruchsvolle Hörsituationen im Störgeräusch (OlSa: Messkonfiguration S_0_N_0_ und S_0_N_HG_) ergab sich ein signifikanter Unterschied: die Altersgruppe der über 70-Jährigen erzielte im Durchschnitt einen weniger negativen Wert im OlSa, was für ein schlechteres Ergebnis für das Verstehen im Störgeräusch spricht. In der Publikation von Lenarz et al. konnte ein ähnlicher Effekt dargestellt werden. Die Autoren nennen als Begründung die zentrale Presbyakusis [13, 15]. Selbst bei klinisch normalhörenden älteren Menschen ist die Sprachwahrnehmung bei verschiedenen Hintergrundgeräuschen schlechter als bei jüngeren Menschen. Als Begründung werden kognitive Faktoren wie das Arbeitsgedächtnis, das eine wichtige Rolle für das SV spielt, genannt [16].

Die Analyse des Zusammenhangs zwischen der Ertaubungsdauer vor CI-Implantation und dem SV in Ruhe im FBE nach CI-Versorgung ergab signifikante Unterschiede. Eine kürzere Ertaubungsdauer zeigte bessere Ergebnisse im SV. Zwischen dem Zeitintervall < 5 Jahre und dem Zeitintervall 5 bis 9 Jahre ergaben sich keine signifikanten Unterschiede. Ein Zeitintervall zwischen Ertaubung und Cochleaimplantation von mehr als 10 Jahren ergab jedoch ein signifikant schlechteres Ergebnis. Die Einteilung des SV in drei Performancegruppen (Abb. 4 rechts) unterstützt diesen Zusammenhang. Die Ertaubungsdauer der sehr guten Performer (61–100 % im FBE) unterschied sich signifikant von den mittleren Performern (30–60 % im FBE) für 80 dB SPL. Der Median der Ertaubungsdauer der sehr guten Performern lag für einen Sprachschallpegel von 65 dB SPL bei 5 Jahren (80 dB SPL: 7 Jahren) und für die mittleren Performer bei 10 Jahren (80 dB SPL: 38 Jahren). Zwischen 10–19 Jahren und > 20 Jahren konnte kein signifikanter Unterschied nachgewiesen werden, ebenso wie zwischen den mittleren (30–60 % im FBE) und schlechten Performern (0–29 % im FBE). Holden et al. konnten feststellen, dass die Dauer des Hörverlusts hoch signifikant und negativ mit der Ergebnisgruppe zusammenhängt [11]. Eine Publikation von Rader et al. konnte bei einer Gruppe einseitig ertaubter und auf der Gegenseite normalhörender Versuchspersonen eine signifikante Korrelation zwischen den audiometrischen Ergebnissen und der anamnestischen Ertaubungsdauer nach 12–36 Monaten nachweisen. Als Gütekriterium der Korrelation wurde das r nach Pearson angegeben, das bei −0,564 lag [24]. In der hier vorliegenden Auswertung wurde als Gütemaß das rho nach Spearman herangezogen. Dieses lag für einen Sprachschallpegel von 65 dB SPL bei −0,404 und für 80 dB SPL bei −0,466, was als mittel einzustufen ist.

Für die hier untersuchte bimodale Versuchspersonengruppe konnte ein Zusammenhang zwischen der anamnestisch erhobenen Ertaubungsdauer und dem CI-Outcome bestätigt werden. Für die klinische Praxis sollte bei Patient:innen, die deutlich länger als 10 Jahre taub sind, dieser Zusammenhang bedacht werden und in die Beratung einbezogen werden, um eine möglichst realistische Erwartungshaltung zu schaffen. Die Auswertung des bimodalen Nutzens war mit zehn Prozentpunkten über den gesamten Untersuchungszeitraum von 36 Monaten über alle Versuchspersonen (unabhängig ihrer Ertaubungsdauer) positiv. Erfreulicherweise stellte sich dieser Nutzen im Median auch schon ganz zu Beginn der Versorgung ein.

Zu den Limitationen der vorliegenden Studie zählt, dass die Ertaubungsdauer lediglich auf Grundlage der in der Anamnese angegebenen Angaben definiert werden konnte, da nicht von einer ausreichenden Anzahl an Patient:innen Audiogramme vor dem Ertaubungszeitpunkt vorlagen. Die Sprachverständlichkeitsmessungen wurden im Alltagsprogramm mit HG und CI durchgeführt, der Einfluss von beispielsweise Störgeräuschunterdrückungen oder Richtmikrofonen stellt daher eine Limitation dar. Da für diese Patient:innen das maximale SV im FBE über Kopfhörer gemessen (SV_max_) nicht systematisch erfasst wurde, konnten die Daten nicht hinsichtlich des Erreichens des Versorgungsziels ausgewertet werden.

## Ausblick

Nach der bimodalen CI-Versorgung sind innerhalb der ersten zwölf Monate deutliche Verbesserungen im SV nachweisbar. Die Verbesserung ist anschließend kleinschrittiger, aber findet dennoch statt, was das Konzept einer therapeutischen Begleitung der CI-Träger:innen über mehrere Jahre hinweg unterstützt. Für den Einflussfaktor Alter bei Versorgung konnte für die Messungen in Ruhe kein Unterschied nachgewiesen werden, was eine Cochleaimplantation auch im fortgeschrittenen Alter unterstützt. Der Grad der Schwerhörigkeit des Gegenohrs spielte ebenfalls keine Rolle; alle Patientinnen und Patienten profitierten unabhängig vom Hörverlust in gleichem Maße von der Versorgung mit dem CI. Wie in vielen anderen Arbeiten stellte auch hier die Ertaubungsdauer einen negativen Einflussfaktor für das SV mit dem CI dar.

## Fazit für die Praxis

- Das SV verbessert sich in Ruhe und im Störgeräusch im zeitlichen Verlauf nach der Cochleaimplantation bei der bimodalen Versuchspersonengruppe signifikant.
- Die Ertaubungsdauer ist ein negativer Einflussfaktor für das SV mit dem CI und sollte bei der Beratung bimodaler Kandidat:innen beachtet werden, um eine möglichst realistische Erwartungshaltung zu schaffen.
- Die Einflussfaktoren Alter bei Versorgung und Grad der Schwerhörigkeit des Gegenohrs hat keinen Einfluss auf das SV in Ruhe, was die aktuelle Indikationsstellung in Deutschland unterstützt – auch in fortgeschrittenem Alter und/oder bei geringgradiger Schwerhörigkeit der Gegenseite eine Cochleaimplantation durchzuführen.
- Der bimodale Nutzen war über den gesamten Untersuchungszeitraum im Median positiv und zeigte, dass sich für knapp 80 % der Versuchspersonen trotz notwendiger Integration von akustischer und elektrischer Information die bimodale Versorgung als vorteilhaft erwies.

## Data Availability

Die erhobenen Datensätze sind in anonymisierter Form bei der korrespondierenden Autorin auf Anfrage verfügbar.
